# Diagnostic Tools Used for Tuberculous Pleural Effusion and Their Association With Treatment Outcomes

**DOI:** 10.7759/cureus.118376

**Published:** 2026-10-22

**Authors:** Afraa Mohammed, Elrasheid Ali Awouda, Ashwag Elbadri, Ahmed Abuelgasim

**Affiliations:** 1 Gastroenterology and Hepatology, Sudan National Center for Gastrointestinal and Liver Diseases, Khartoum, SDN; 2 Pulmonary Medicine, Shaab Teaching Hospital, Khartoum, SDN; 3 Oncology, King Abdulaziz Hospital, Jeddah, SAU; 4 Medicine, Joseph Wright Centre, Derby College Group, Derby, GBR

**Keywords:** adenosine deaminase, diagnostic testing, gene xpert, pleural fluid, sudan, treatment outcome, tuberculosis, tuberculous pleural effusion

## Abstract

Background

Tuberculous pleural effusion (TPE) is difficult to confirm because pleural disease is often paucibacillary. Pleural-fluid biomarkers, culture, molecular assays, and pleural biopsy can improve diagnostic certainty, but their availability varies substantially in resource-limited settings.

Objective

To describe the diagnostic investigations used for suspected TPE at AlShaab and Abu Anjaa Teaching Hospitals in Khartoum, Sudan, from February to July 2017, and assess their association with treatment outcomes.

Methodology

This retrospective cohort study included 69 patients with a clinician-recorded diagnosis of TPE at two teaching hospitals in Khartoum, Sudan, from February to July 2017. The first author collected demographic, clinical, investigation, treatment, and outcome data from patient records using a standardized data-collection checklist. Investigations were classified according to whether a test and result were documented. Associations with outcome were assessed using Fisher’s exact test and odds ratios (ORs) with 95% confidence intervals (CIs).

Results

Chest radiography was performed in 67/69 patients (97.1%). Pleural-fluid adenosine deaminase (ADA) was recorded in 1/69 (1.4%), culture in 6/69 (8.7%), polymerase chain reaction (PCR) in 4/69 (5.8%), and pleural biopsy in 7/69 (10.1%). GeneXpert MTB/RIF (Cepheid, Sunnyvale, CA) and pleural-fluid interferon-gamma testing were not performed. HIV testing was not documented in 50/69 patients (72.5%). Thirteen patients (18.8%) received antituberculous treatment without pleural-fluid or tissue testing. Five (38.5%) had a favorable outcome, and eight (61.5%) had an unfavorable outcome, including four subsequently diagnosed with malignant effusion and one death. Among 56 patients who underwent at least one pleural-fluid or tissue test, 39 (69.6%) had a favorable outcome. Any diagnostic testing was associated with higher odds of favorable outcome (OR 3.67, 95% CI 1.05-12.87; Fisher's exact *P* = 0.054), although the association did not cross the conventional statistical-significance threshold.

Conclusions

Confirmatory testing for suspected TPE was used inconsistently, while several clinically useful tests were rarely or never available. Patients treated without pleural-fluid or tissue testing had more unfavorable outcomes, including missed malignant effusion. The findings support a pragmatic minimum diagnostic bundle before prolonged empiric treatment, while recognizing that this observational study cannot establish that testing caused better outcomes.

## Introduction

Tuberculous pleural effusion (TPE) is a common extrapulmonary manifestation of tuberculosis and remains difficult to diagnose because the pleural space often contains few viable organisms. A recent clinical review describes tuberculous pleural effusion (TPE) as a paucibacillary disease in which pleural-fluid biomarkers and cartridge-based polymerase chain reaction (PCR) can contribute. However, pleural biopsy may still be required when fluid studies are indeterminate [1]. The diagnostic problem is especially consequential in settings where treatment may be initiated empirically and alternative causes of pleural effusion, including malignancy, are not systematically excluded.

Pleural-fluid adenosine deaminase (ADA) and interferon-gamma are the best-established biochemical markers. In a comparative meta-analysis of 45 paired datasets from 44 publications involving 4,974 patients, pleural-fluid ADA had pooled sensitivity and specificity of 0.88 and 0.91, respectively, while interferon-gamma had pooled sensitivity and specificity of 0.91 and 0.96 [2]. The same analysis favored interferon-gamma over ADA, although it also found substantial heterogeneity, variable thresholds, and a high risk of bias in most contributing studies [2]. ADA remains attractive in low-resource settings because it is relatively inexpensive and widely available; however, its interpretation depends on the clinical context and local prevalence.

Direct microbiological testing is useful but limited by low organism burden. A prospective comparison of pleural-fluid ADA, liquid culture, sputum testing, and pleural biopsy found pleural-fluid ADA at a threshold of 50 U/L in 44/46 patients with tuberculosis (TB), pleural-fluid Mycobacteria Growth Indicator Tube (MGIT) culture in 31/46, and pleural biopsy in 44/49 [3]. Automated liquid culture can improve yield and shorten time to positivity compared with solid media: in one study, MGIT culture was positive in 40.4% of samples compared with 18.3% for Ogawa medium, and median time to positivity was 18 versus 37 days [4].

Molecular testing has a different role. A systematic review concluded that Xpert MTB/RIF (Cepheid, Sunnyvale, CA) on pleural fluid has limited value as a screening test but high potential to confirm pleural TB when positive [5]. Thus, a negative pleural-fluid molecular result should not be used alone to exclude TPE. Pleural tissue may provide a higher-yield specimen when pleural endoscopy or biopsy is available; in a 2022 pleuroscopy cohort, culture sensitivity was 34.8% for pleural fluid and 78.3% for pleural tissue [6].

HIV coinfection may affect the likelihood of microbiological confirmation in pleural TB. A systematic review found that pleural-fluid smear and culture yields may be higher in people with HIV than in those without HIV, while ADA and interferon-gamma remain useful markers in both groups [7]. Another study also reported a higher proportion of positive pleural-fluid cultures among HIV-positive patients [8]. Pleural-fluid neutrophilia and loculation have also been associated with greater mycobacterial burden and higher diagnostic yield [9].

Sudan has a substantial burden of extrapulmonary TB. In a hospital-based study from Kassala, 224 of 985 patients with TB (22.7%) had extrapulmonary disease; diagnostic classification used histology, PCR, imaging, fluid findings, and clinical response [10]. However, evidence describing the real-world diagnostic pathway for suspected TPE in Sudanese hospitals is limited. This study therefore assessed which diagnostic tools were used in two Khartoum hospitals and whether testing was associated with six-month treatment outcome.

## Materials and methods

Study design and setting

This retrospective cohort study was conducted at AlShaab Teaching Hospital and Abu Anjaa Teaching Hospital in Khartoum, Sudan. Data were collected from February to July 2017. Abu Anjaa Teaching Hospital was a specialist center for multidrug-resistant tuberculosis (MDR-TB).

Study population and case definition

Patients were eligible if the treating clinician had recorded a diagnosis of TPE during the study period. Patients with pulmonary TB without pleural involvement and those with a confirmed nontuberculous cause of pleural effusion were excluded. In this setting, TPE was often diagnosed clinically because many patients could not afford further investigations and several pleural-fluid biomarkers were expensive or not widely available. The recorded diagnosis reflected the treating clinician’s overall assessment, which could include clinical features, radiography, pleural-fluid findings when available, and the local TB epidemiological context. No uniform clinical or radiological criteria or microbiological or histological reference standard was applied, and the retrospective records did not contain sufficiently consistent information to apply standardized criteria. All 69 eligible patients identified during the study period were included. Because this was a retrospective review, the records did not show how many patients were screened or excluded before the 69 eligible patients were identified.

Sample size calculation

Referral-clinic records from February to July 2017 included 646 TB-related attendances among 3,079 total attendances (21.0%). Using the formula:

$n=\frac {Z^2pq}{d^2}$.

With *Z *= 1.96, *p *= 0.21, *q *= 0.79, and *d *= 0.0955, the estimated minimum sample size was 69.9. We included all 69 eligible patients who presented during the study period. The study was therefore a time-bound convenience cohort and was not powered for individual diagnostic-test comparisons.

Data collection

The first author collected the data from the available patient records using a standardized data-collection checklist. The checklist captured demographic information, medical history, investigations, diagnosis, treatment, and outcome.

Diagnostic testing exposure

For each diagnostic test, exposure was defined as the test being performed and having a recorded result. Patients were also classified according to whether at least one pleural-fluid or tissue investigation had been performed and its result documented. These investigations included pleural-fluid culture, PCR, ADA, cytology, Ziehl-Neelsen staining, and pleural biopsy. This composite measure was used solely to describe whether any such investigation was documented; it does not imply that the tests were equivalent or assess their individual diagnostic performance. Chest radiography and routine blood tests were not included in the composite measure.

Treatment and outcome assessment

Patients were treated according to the national TB treatment guidelines in Sudan, rather than a separate hospital-specific guideline. Abu Anjaa Teaching Hospital specialized in drug-resistant TB; therefore, patients treated for drug-resistant TB received different antituberculous regimens in accordance with the national guidance. Regimens were consequently not uniform across the cohort. Favorable response was defined pragmatically by documented clinical or radiological improvement, including resolution of fever, normalization of ESR, or resolution of pleural effusion. Final outcome was classified as resolved, malignant effusion, unresolved effusion, incomplete resolution, empyema requiring surgery, or death.

Statistical analysis

Categorical data are presented as numbers and percentages. Final outcome was recorded for all 69 patients. Other variables were analyzed using the information available in the medical records, and missing values were not imputed. For each diagnostic test, we compared patients whose records included a result with those whose records did not. A missing result was not taken to mean that the test had not been performed.

We used Fisher’s exact test to assess the association between documented test results and fever resolution, ESR normalization, effusion resolution, and final outcome, because some expected cell counts were small. Odds ratios (ORs) and 95% confidence intervals (CIs) were calculated. When a cell count was zero, we applied the Haldane-Anscombe correction. All tests were two-sided, with P < 0.05 considered statistically significant. As the sample was small and several exploratory comparisons were made, P-values were interpreted with caution. Analyses were performed using IBM SPSS Statistics, version 21 (IBM Corp., Armonk, NY) and Python with SciPy.

Ethical considerations

The study protocol was approved by the Ethics Review Committee of the Sudan Medical Specialization Board, Council of Internal Medicine. Permission was obtained from both hospitals.

## Results

Baseline characteristics

Sixty-nine patients were enrolled. Thirty-three patients (47.8%) were aged 20-39 years, 50 (72.5%) were male, and 19 (27.5%) were female. Twenty-five patients (36.2%) lived outside Khartoum State. The most frequent symptoms were cough in 41 patients (59.4%), weight loss in 39 (56.5%), fever in 35 (50.7%), pleuritic chest pain in 31 (44.9%), and shortness of breath in 26 (37.7%). Symptom duration exceeded four weeks in 61 patients (88.4%).

Diagnostic testing

Chest radiography was performed in 67/69 patients (97.1%). Mantoux testing was not documented in 37 patients (53.6%). Pleural-fluid ADA was recorded in 1 patient (1.4%), culture in 6 (8.7%), PCR in 4 (5.8%), and pleural biopsy in 7 (10.1%). GeneXpert MTB/RIF and pleural-fluid interferon-gamma testing were not documented. HIV testing was not documented in 50 patients (72.5%). The diagnostic tests performed are summarized in Table 1.

**Table 1:** Diagnostic tests performed in 69 patients with suspected TPE. TPE, tuberculous pleural effusion; ADA, adenosine deaminase; HIV, human immunodeficiency virus; MTB, Mycobacterium tuberculosis; PCR, polymerase chain reaction; RIF, rifampicin

Test	Performed, *n* (%)	No result documented, *n* (%)
Chest radiograph	67 (97.1)	2 (2.9)
Mantoux test	32 (46.4)	37 (53.6)
Pleural-fluid ADA	1 (1.4)	68 (98.6)
Pleural-fluid culture	6 (8.7)	63 (91.3)
Pleural-fluid PCR	4 (5.8)	65 (94.2)
Pleural biopsy	7 (10.1)	62 (89.9)
GeneXpert MTB/RIF	0 (0.0)	69 (100.0)
Pleural-fluid interferon-gamma	0 (0.0)	69 (100.0)
HIV test	19 (27.5)	50 (72.5)

Treatment response

Fever resolved during the first month in 22 patients (31.9%) and between one and three months in a further 31 patients (44.9%); it persisted in 16 patients (23.2%). ESR normalized within six months in 28 patients (40.6%) and remained abnormal in 41 (59.4%). Pleural effusion resolved within three months in 31 patients (44.9%) and after three months in a further 13 (18.8%); it persisted in 25 patients (36.2%).

Final outcome was recorded as resolved in 44 patients (63.8%). Other outcomes were malignant effusion in eight (11.6%), unresolved effusion without a specific complication in five (7.2%), incomplete resolution in four (5.8%), empyema requiring surgery in four (5.8%), and death in four (5.8%).

Association between diagnostic testing and outcome

Thirteen patients (18.8%) received antituberculous treatment without any pleural-fluid or tissue diagnostic test. Of these 13 patients, five (38.5%) had a favorable response, whereas eight (61.5%) had an unfavorable outcome. The unfavorable outcomes included malignant effusion in four patients, empyema in one, incomplete resolution in one, unresolved effusion of uncertain cause in one, and death in one.

Among the 56 patients who underwent at least one pleural-fluid or tissue test, 39 (69.6%) had a favorable outcome and 17 (30.4%) did not. Any pleural-fluid or tissue testing was associated with higher odds of favorable outcome than no such testing (OR 3.67, 95% CI 1.05-12.87; Fisher's exact *P* = 0.054). The point estimate favored testing, but the association did not meet the prespecified *P* < 0.05 threshold. Associations between diagnostic investigations and treatment outcomes are summarized in Table 2.

**Table 2:** Association between diagnostic testing and selected outcomes. CI, confidence interval; ESR, erythrocyte sedimentation rate; OR, odds ratio; PCR, polymerase chain reaction

Exposure	Outcome assessed	Association	Interpretation
Any pleural fluid or tissue test versus none	Favorable final outcome	OR 3.67, 95% CI 1.05-12.87; *P* = 0.054	Higher odds with testing; imprecise and narrowly non-significant
Mantoux test versus not documented	Fever resolution	*P* = 0.253	No statistically significant association
Mantoux test versus not documented	ESR normalization	*P* = 0.633	No statistically significant association
Mantoux test versus not documented	Effusion resolution	*P* = 0.219	No statistically significant association
PCR versus not documented	Fever resolution	*P* = 0.566	No statistically significant association; very small tested subgroup
PCR versus not documented	ESR normalization	*P* = 1.000	No statistically significant association; very small tested subgroup
PCR versus not documented	Effusion resolution	*P* = 0.289	No statistically significant association; very small tested subgroup
Pleural biopsy versus not documented	Fever resolution	*P* = 1.000	No statistically significant association; very small tested subgroup
Pleural biopsy versus not documented	ESR normalization	*P* = 0.228	No statistically significant association; very small tested subgroup
Pleural biopsy versus not documented	Effusion resolution	*P* = 0.043	Exploratory association; requires cautious interpretation because of sparse data and multiple comparisons

## Discussion

This study describes a substantial gap between the diagnostic tools supported by the literature and those used in two Sudanese hospitals. Chest radiography was nearly universal, but pleural-fluid ADA, culture, PCR, and biopsy were uncommon, while GeneXpert MTB/RIF and interferon-gamma testing were not used. The most clinically concerning finding was the group treated without any pleural-fluid or tissue diagnostic test: it represented 18.8% of the cohort, had a lower proportion of favorable outcomes, and included four patients later diagnosed with malignant effusion.

The underuse of ADA is notable because pleural-fluid ADA has a strong evidence base and is relatively accessible compared with thoracoscopy or interferon-gamma testing. Comparative evidence suggests that interferon-gamma is somewhat more accurate than ADA, but the difference must be weighed against cost, laboratory requirements, and availability [2]. In a low-resource setting, ADA may therefore be a practical first-line biomarker, while interferon-gamma testing or tissue sampling can be reserved for diagnostically uncertain cases.

The low use of culture and PCR also matters because a microbiologically confirmed diagnosis can support drug-susceptibility testing and help distinguish TPE from malignancy or other inflammatory effusions. However, negative pleural-fluid results should not be interpreted as exclusionary. Pleural TB is often paucibacillary, and Xpert MTB/RIF has limited sensitivity when used as a pleural-fluid screening test [5]. The most defensible interpretation of a positive molecular result is confirmation; a negative result should prompt consideration of ADA, sputum testing, pleural biopsy, or another appropriate diagnostic pathway.

The evidence also supports a stepwise approach to specimen selection. Liquid culture may outperform conventional solid media [4], and pleural tissue obtained during pleuroscopy may have greater culture sensitivity than pleural fluid [6]. The biological context is important: HIV coinfection, pleural-fluid cellular composition, and loculation may affect organism burden and microbiological yield [7]. These factors should be measured and reported in future Sudanese cohorts rather than treating a negative fluid culture as evidence against TPE.

The association between any diagnostic testing and favorable outcome is clinically plausible but should not be interpreted causally [11]. Patients who received testing may have differed from untested patients in referral pathway, clinician concern, disease severity, ability to pay, access to procedures, or likelihood of receiving closer follow-up. Conversely, testing may have been preferentially ordered for patients with more severe or atypical disease. The OR of 3.67 is therefore best understood as a signal that diagnostic workup and outcome were related in this cohort, not as an estimate of the treatment benefit caused by testing.

Four of the 13 patients without a documented pleural-fluid or tissue investigation were later diagnosed with malignant effusion. This finding highlights the importance of considering alternative diagnoses when assessing suspected TPE, particularly when the effusion persists or has atypical features. Cytology and further assessment may help identify these alternatives. Because investigations were not applied systematically and no uniform reference standard was used, this study cannot determine the sensitivity or specificity of cytology, ADA, PCR, or pleural biopsy.

Study limitations: Diagnostic tests were not performed uniformly; their use depended on clinical need and availability, and some tests were performed in very few patients. The composite measure therefore shows only whether any pleural-fluid or tissue investigation was documented. It combines tests with different purposes, including cytology, which can help identify other causes such as malignancy. It should not be viewed as a single diagnostic approach or used to compare the performance of individual tests. In addition, this was a retrospective review of records from 2017 at two teaching hospitals in Khartoum. The findings may not reflect current practice or apply to other settings.

## Conclusions

In this cohort from two hospitals in Khartoum, investigations for suspected TPE were not consistently documented. Patients without a documented pleural-fluid or tissue investigation had fewer favorable outcomes, and four were later found to have malignant effusions. In similar settings, a practical diagnostic workup could include pleural-fluid biochemistry and cytology, ADA and TB testing where available, sputum testing when pulmonary TB is suspected, and pleural biopsy or thoracoscopy if an effusion persists or remains atypical. These findings support considering a locally feasible diagnostic pathway, but this small observational study cannot show that testing improved outcomes. Prospective studies using consistent diagnostic criteria and outcome definitions are needed.
